# Chronic stress enhances glycolysis and promotes tumorigenesis

**DOI:** 10.3389/fonc.2025.1543872

**Published:** 2025-03-10

**Authors:** Qiufeng Qin, Shuying Li, Yixuan Zhong, Jing Bai, Lin An, Lei Yang, Wei Gu, Di Deng, Jinlan Zhao, Rong Zhang, Haiquan Liu, Shasha Bai

**Affiliations:** ^1^ From the School of Pharmaceutical Science, International Institute for Translational Chinese Medicine, Guangzhou University of Chinese Medicine, Guangzhou, China; ^2^ Pharmacy Department, JiNan Authority Hospital, Jinan, China; ^3^ Huizhou Hospital of Guangzhou University of Chinese Medicine/Huizhou Hospital of Traditional Chinese Medicine, Huizhou, China

**Keywords:** CUMS, metastasis, tumorigenesis, glycolysis, orthotopic

## Abstract

Depression is a well-known risk factor for tumors, but the mechanisms other than inflammation are unclear. Aerobic glycolysis is considered to be a critical element in the reprogramming of energy metabolism in malignant tumors, and impaired glycolysis has been reported in the brains of chronic stress mice. Therefore, this study aimed to explore the role of glycolysis in which depression promotes tumorigenesis. We examined the impacts of chronic unpredictable mild stress (CUMS) on the growth and metastasis of breast cancer (BC) and lung cancer (LC). CUMS was used to construct a mouse depression model, BALB/c mice were injected with 4T1-Luc cells in the right subcutaneous mammary fat pad, and C57BL/6 mice were injected with Lewis-Luc cells in the tail vein. The experiments were conducted through behavioral experiments, live imaging techniques of small animals, Western blot, Glycolytic metabolites measurement, Hematoxylin and eosin staining (H&E staining), Nissl staining, and immunohistochemical (IHC) tests. The findings showed that both CUMS and tumors induced depressive-like behavior, neuronal damage, and impaired synaptic plasticity in mice, while CUMS also enhanced tumor development and metastasis in both BC and LC. In the brain, both CUMS and tumor alone and in combination less influence glycolytic products and enzyme levels. However, CUMS significantly enhanced the levels of aerobic glycolytic products and enzymes in tumor tissue. Collectively, our results provide insights into how glycolysis is regulated in the brain, leading to depression-like behavior, and how depression, in turn, enhanced glycolysis and promoted tumorigenesis.

## 1 Introduction

Patients diagnosed with cancer often concurrently suffer from depression (1, 2). The negative emotions induced by this psychological condition can potentially stimulate tumor growth and metastasis of tumors (3–5). Past studies have unequivocally shown that chronic stress significantly affects every stage of cancer development in patients, ranging from carcinogenesis to angiogenesis stimulation and metastatic dissemination (6). Chronic stress-induced inadequate coping, negative emotional responses, or a diminished quality of life are associated with an increased risk of cancer incidence (7). Annually, approximately one million new cancer cases are diagnosed in young individuals aged 20-39 years, primarily attributed to chronic stress (8). A poor prognosis and elevated mortality are observed among cancer patients concurrently suffering from comorbid depression (6). Cancer patients exhibit a depression morbidity of approximately 12.5%, which is up to fourfold higher than that of the general population (9). Chronic stress has been demonstrated in studies to raise plasma catecholamine levels, including epinephrine and noradrenaline, and accelerate the aggressive development of BC, ovarian carcinoma, and gastric cancer (10, 11). However, the research concerning the relationship between chronic stress and cancer remains limited.

Otto Warburg and colleagues ascertained in the 1920s that tumor cells, under aerobic conditions, exhibit a preference for generating energy via a process termed aerobic glycolysis. This is manifested by excessive glucose absorption and lactate accumulation, a phenomenon known as the Warburg effect (12, 13). To fulfill uncontrolled biosynthesis and energy demands, cancer cells frequently tend to perform aerobic glycolysis (13). In numerous cancer patients, clinical investigations utilizing the imaging technique of positron emission tomography (PET) with the glucose analog tracer 18 fludeoxyglucose (FDG) 6-8 have unambiguously demonstrated a substantial elevation in glucose absorption in the majority of metastatic and primary human cancer patients (14). Tumor cells facilitate the formation of new blood vessels through the secretion of VEGF, induced by lactate, which supplies sufficient oxygen and nutrients for proliferation (15). Persistent chronic stress triggers a decrease in lactate levels and leads to depression. The “Warburg” effect occurs not only in tumor cells but also in non-tumor cells (16). Lactic acid acts as a signaling molecule involved in the regulation of brain function; both a deficiency and accumulation of lactic acid can lead to neurological dysfunctions, such as depression (17). Under physiological conditions, aerobic glycolysis in the brain contributes to dendritic growth, myelination processes, as well as the activities of neurons and microglia, while also reducing oxidative stress (18, 19). Intriguingly, stress-induced epinephrine enhances breast cancer stem-like characteristics through LDHA (lactate dehydrogenase A) - dependent metabolic reprogramming (11). Lactate performs antidepressant functions by maintaining normal neuron function, and modulating levels and activity of histone deacetylases in the hippocampal region (20). Meanwhile, LDHA regulates neuronal excitability to inhibit depressive-like behavior through lactate homeostasis (21). However, the research on lactate levels in depression and cancer models has been a gap in the literature. To address this, we developed an animal model of breast cancer-related depression (BCRD) by *in situ* injection of BC cells into CUMS mice and a model of lung cancer-related depression (LCRD) by injecting LC cells into the tail veins of CUMS mice. Behavioral assessments and synapse plasticity detection were used for depression evaluation, while tumor volume and H&E staining were performed to examine tumorigenesis. Metabolites and key functional enzymes were detected to unreal the role of glycolysis in depression-promoted tumor glycolysis.

The findings of this research highlight the potential role of chronic stress in exacerbating tumorigenesis and metastasis through the stimulation of glycolysis.

## 2 Materials and methods

### 2.1 Cell culture

Luciferase gene-tagged Lewis murine lung cancer cell (LLC-Luc) and luciferase gene-tagged 4T1 murine breast cancer cell (4T1-Luc) were purchased from the Chinese Academy of Sciences Cell Bank (Shanghai, China). LLC-Luc cells were cultured in Dulbecco’s modified Eagle’s medium (DMEM, Gibco-BRL, Grand Island, NY, USA); the 4T1-Luc cells were cultured in RPMI-1640 medium (Gibco-BRL, Grand Island, NY, USA); all mediums were supplemented with 10% fetal bovine serum and cultured at 37°C in a humidified incubator containing 5% CO_2_.

### 2.2 Animals

Six–week–old female BALB/c mice and male C57BL/6 mice were purchased from the Guangdong Medical Laboratory Animal Center (Guangdong, China), and housed in special-pathogen-free ventilation facilities. 12 h/12 h light/dark cycle was carried out, with ambient temperatures of 20 - 26° C and relative humidity of 40 – 70%, 5 mice per cage, and eating and drinking freely. The Ethics Committee of Guangzhou University of Chinese Medicine has authorized a laboratory animal protocol.

### 2.3 Chronic unpredictable mild stress experiment

Chronic unpredictable mild stress (CUMS) might mimic the onset of depression produced by various pressures in human daily life (5, 22). Mice were exposed to CUMS for 8 weeks, which included day-night reversal (24 h), cold-water swimming (10°C ± 1°C, 3 min), crowd-feeding (24 h), water and food deprivation (24 h), an empty water bottle (24 h), a 45°cage tilt (24 h), a tail clamp (1 cm from the tail end, 60 s), a self-made plastic seal tube (3 h), and a wet pad (24 h). 2 or 3 stimulation modalities were chosen randomly each day to ensure no repetition within 2 days.

### 2.4 Establishment of tumor orthotopic transplantation

5 × 10^5^ 4T1-Luc cells were injected into the right second mammary fat pad of BALB/c mice for orthotopic transplantation of BC. CUMS mice were inoculated with 4T1-Luc cells to develop BCRD. The tumor size of BC was measured with a vernier caliper every two days. It was calculated as follows: tumor volume (mm^3^) = [length × width^2^]/2.

1 × 10^6^ LLC-Luc cells were injected into the tail vein of C57BL/6 mice for orthotopic transplantation of LC, and CUMS mice adopted LLC-Luc cells to develop LCRD. The tumor size was evaluated every week using IVIS (PerkinElmer, Boston, United States) with 150 mg/kg of D-luciferin potassium salt (PerkinElmer, Boston, United States) given intraperitoneally.

### 2.5 Behavior assessments

The sucrose preference test (SPT) is done for anhedonia assessment. Mice were fed in solitary cages preferentially. Two bottles containing 1% sucrose solution were placed at each side of the cages for 24 h as an adaption phase. The next day, one bottle was replaced with water and left for another 24 h, and two bottles of solution were changed the position at 12 h to avoid the error caused by location preference. Then, mice were formally tested after 24 h of freely eating without water, one bottle of water and one bottle of 1% sucrose solution were placed on each side of the cage and switched at 12 h. 24 h later, weigh each bottle and calculate sucrose preference (%) = sucrose consumption/(water consumption + sucrose consumption) × 100% (23).

An open field test (OFT) was also performed. BALB/c mice were set up in the center of the blackboard, while C57BL/6 mice were on a whiteboard and 10 minutes for each mouse. The open-field arena is 50 × 50 × 40 cm (length × width × height). The mice’s activity was filmed using a digital camera, and the total distance, center distance, and center time were calculated using software.

The tail suspension test (TST) was done as follows, mice were suspended 30 cm above the ground with wide tape on the tail-suspension device for 6 minutes and recorded with a digital camera recorded the mice’s activity. The first 2 minutes are considered familiarity time and only the last 4 minutes are counted.

Mice were placed in cylindrical containers filled with room-temperature water at a depth of 30 cm for forced swim test (FST). Each mouse swam for 6 minutes and was videotaped, and then blow-dried the mice hair and put them back. The first 2 minutes are considered familiarity time and only the last 4 minutes are counted.

The Y-Maze test was considered evaluable for working memory and exploratory behavior in mice. Mice were placed on either of three identical arms (arm length: 35 cm, arm width: 5 cm, wall height: 10 cm) and were allowed to explore freely for 8 minutes. The percentage of spontaneous alternation was calculated as alternation (%) = (number of correct alternations)/(number of total arm entries-2) x 100% (24).

The elevated plus-maze test (EPM) was used to investigate anxiety-related behavior in rodents. The maze consists of four arms: a pair of open arms and a pair of closed arms (35 cm long, 5 cm wide, and 10 cm high), which were connected by a central platform. The maze rises 50 cm above the ground. Gently place the mouse in the central area facing the open arm and track the mouse’s movements within the elevated cross-maze instrument. Record the number of entries and the time spent in each arm (25).

### 2.6 Western blot

Tissue protein was extracted with ice RIPA and was detected with a BCA kit following the guidelines. The protein samples were split using SDS-PAGE, moved to a PVDF membrane, and then treated with primary antibodies such as HKII (hexokinase II), PFKP (phosphofructokinase platelet type), PKM2 (pyruvate kinase isozyme type M2), PDH (pyruvate dehydrogenase), and LDHA (Lactate dehydrogenase A) overnight at 4°C. After incubation with enzyme-linked secondary antibody, the target protein level was detected with a super-ECL detection reagent.

### 2.7 Glycolytic metabolites measurement

The contents of the ATP colorimetric kit (Cat.#A095-1-1), pyruvate test kit (Cat.#A081-1-1), and lactic acid assay kit (Cat.#A019-2) were obtained from Nanjing Jiancheng, Nanjing, China and followed the manufacturer’s instructions.

### 2.8 Hematoxylin and eosin staining, Nissl staining

After being fixed with 4% paraformaldehyde for 24 h, the tissue samples were paraffin-embedded and sliced into 4 μm. The tissue sections were deparaffinized with xylene and graded alcohol. According to the standard protocol of H&E staining, eosin dye for 3 minutes and hematoxylin dye for 10 minutes. Standard Nissl’s staining method was performed (26).

### 2.9 Immunohistochemistry

Tissue slices were deparaffinized, and antigen recovery was performed in citrate buffer (pH=6). Then slices were incubated with the specific antibody HKII (AB227198) in the wet box at 4°C overnight. The DAB detection kit was used as a color developer after the secondary antibody was combined, and finally, the percentage of positive cells was estimated with Image J analysis software (27). All images were taken using an optical microscope.

### 2.10 Statistical analysis

All data are presented as mean ± standard error of the mean (SEM). All charts show relevant data for at least 3 independent tests. SPSS 26.0 software was used to analyze the statistical analyses. Student’s t-test was used for between-group comparisons followed by a Tukey *post-hoc* test when an ANOVA revealed significance. A two (± tumor) by two (± CUMS) ANOVA with time as a repeated measure was used for the comparison of four experimental groups. Significance is defined as *P*<0.05 for all analyses.

## 3 Results

### 3.1 CUMS induces depression-like behavior in BALB/c mice and C57BL/6 mice

**Figure 1:**
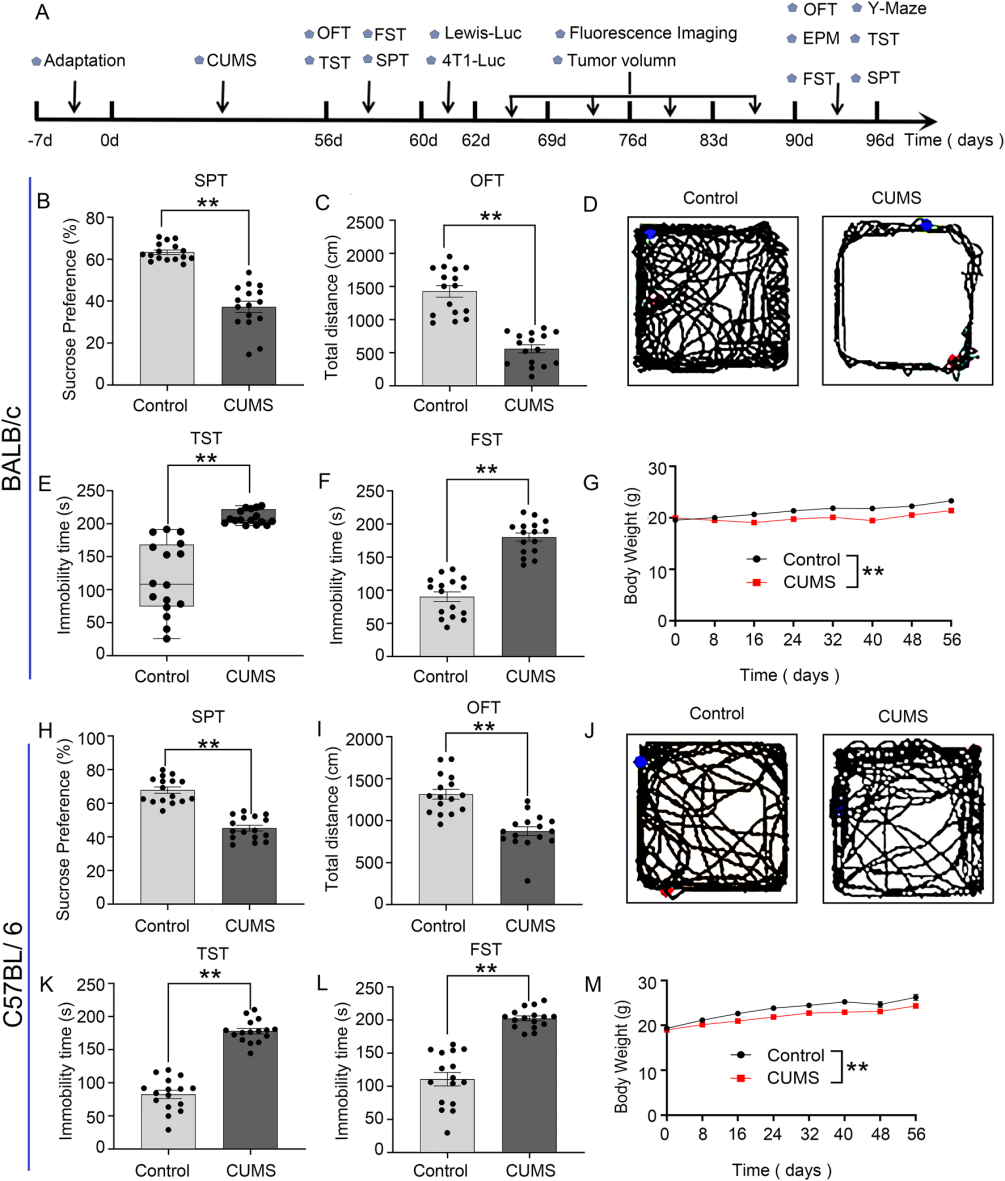
Establishment of the CUMS model in BALB/c mice and C57BL/6 mice: **(A)** Flowchart of the experiment. **(B, H)** CUMS decreased the percentage of sucrose preference in the SPT. **(C, I)** CUMS decreased the total distance in the OFT. **(D, J)** The representative movement trajectories of each group mouse in the OFT. **(E, F, K, L)** CUMS increased immobility time in TST and FST. **(G, M)** The body weight curve of all group mice during the whole experiment. Data are represented as the mean value ± SEM, n = 16, * * p<0.01.

The experimental flow chart is shown in **Figure 1A**. In CUMS group mice, sucrose preference decreased (**Figures 1B, H**), total locomotion diminished in OFT (**Figures 1C, I**), prolonged immobility time (**Figures 1E, F, K, L**), and lower body weights (**Figures 1G, M**) compared to the control group. These results indicate that the depression model was successfully established in BALB/c mice and C57BL/6 mice.

### 3.2 The combination of tumors and CUMS exacerbated depression-like behavior in mice

**Figure 2:**
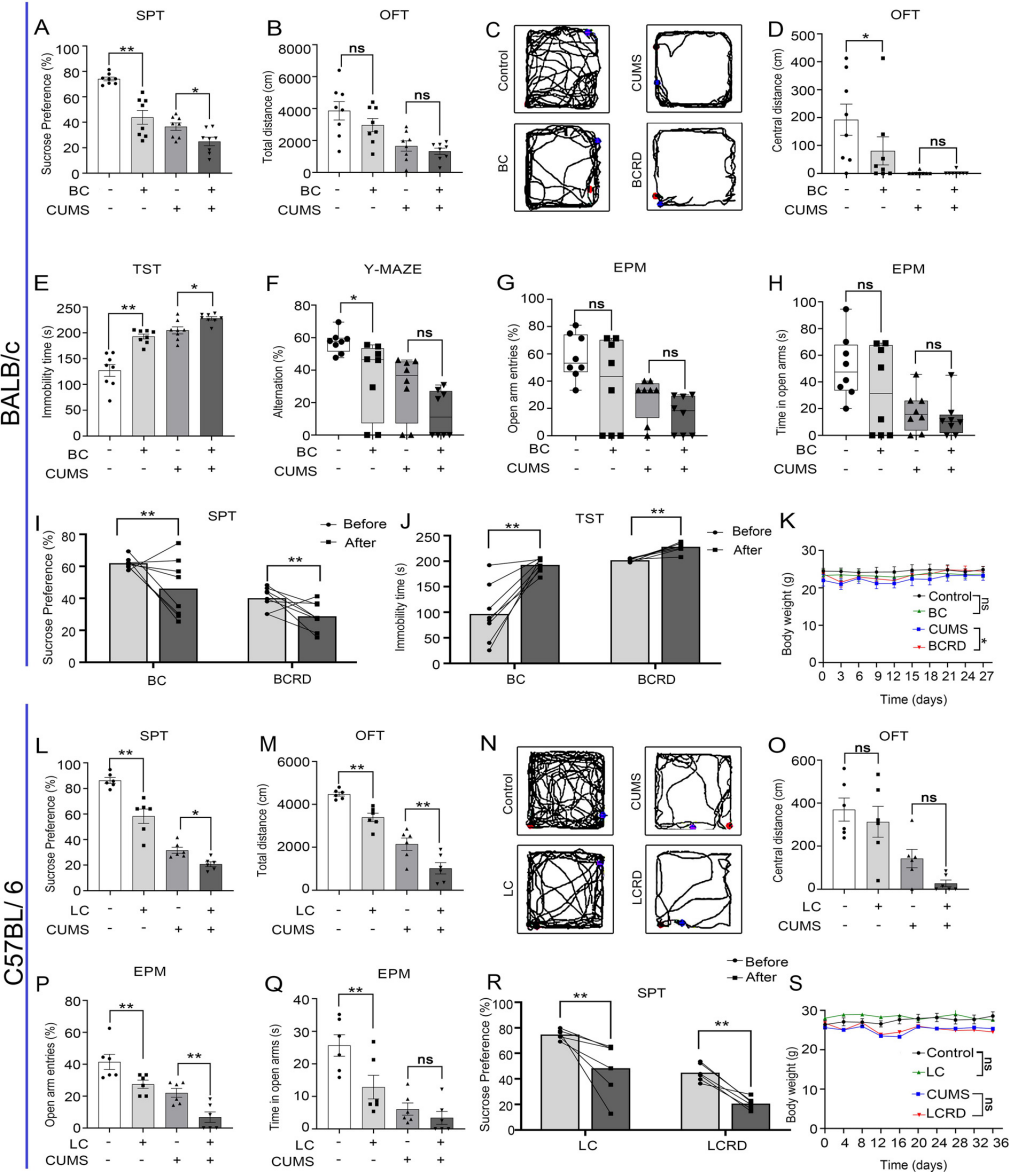
Chronic stress aggravated depression-like behavior in tumor-bearing mice: **(A, L)** The percentage of sucrose preference in the SPT. **(B, M)** Results of the total distance in the OFT. **(C, N)** The representative movement trajectories of each group in the OFT. **(D, O)** Results of the central distance in the OFT. **(E)** Results of immobility time in the TST. **(F)** Results for the number of alternations in the Y-maze. **(G, H, P, Q)** Open arm entry number and residence time in the EPM. **(I, J, R)** Self-behavioral comparison. **(K, S)** The body weight curve. Data are represented as the mean value ± SEM (BALB/c: n= 8, C57BL/6: n= 6), * p<0.05, ** p<0.01. ns, no significant.

To investigate whether the mice in the tumor group exhibited anxiety-like and depressive-like behaviors and whether depression-like behaviors were more severe in the BCRD and LCRD (24), we performed behavioral assessments on tumor-bearing mice separately. Using two-way ANOVA analysis, the results show that in BALB/c mice, tumor-bearing decreased sucrose preference (**Figure 2A**), decreased central activity distance (**Figure 2D**), prolonged immobility time of TST (**Figure 2E**), and impaired spatial cognition (**Figure 2F**) compared to the control group. Compared to the CUMS group, BCRD mice displayed more decreased sucrose preference in SPT (**Figure 2A**), and more prolonged immobility time (**Figure 2E**). No significance in total locomotion in OFT (**Figures 2B, C**), no significance in central activity distance (**Figure 2D**) and spatial cognitive dysfunction (**Figure 2F**), and no significance in the number and stay time of mice entering the open arm between CUMS group and BCRD group (**Figures 2G, H**). Meanwhile, we also assessed the change of SPT and TST of each mouse before and after bearing the tumor. The data showed both control and CUMS mice occurred obvious anhedonia and prolonged immobility time after tumor-bearing, and CUMS induced more individuals and greater variation depression in tumor-bearing mice (**Figures 2I, J**).

In C57BL/6 mice, compared to the control group, tumor-bearing mice exhibited a reduced preference for sucrose (**Figure 2L**), decreased total distance (**Figures 2M, N**), and fewer entries into the open arms, along with reduced dwell time in the open arms (**Figures 2P, Q**) Compared to the CUMS group, LCRD mice displayed more decreased sucrose preference in SPT (**Figure 2L**), more diminished total locomotion in OFT (**Figures 2M, N**), decreased number of mice entering the open arm (**Figure 2P**), no significance in central activity distance (**Figure 2O**). Regardless of whether CUMS was applied or not, the behavior of the lung cancer mice changed, showing a depression-like tendency after tumor loading (**Figure 2R**), which is consistent with what was observed in breast cancer models. Also, the results showed that CUMS stimulation could reduce body weight in mice, consistent with **Figure 1**. Regardless of whether the mice received CUMS stimulation, tumor-bearing had little effect on the body weight of mice. Only the CUMS group and BCRD group exhibited a significant difference in body weight (**Figures 2K, S**).

### 3.3 Chronic stress-induced impairment of hippocampal neurons in tumor-bearing mice

**Figure 3:**
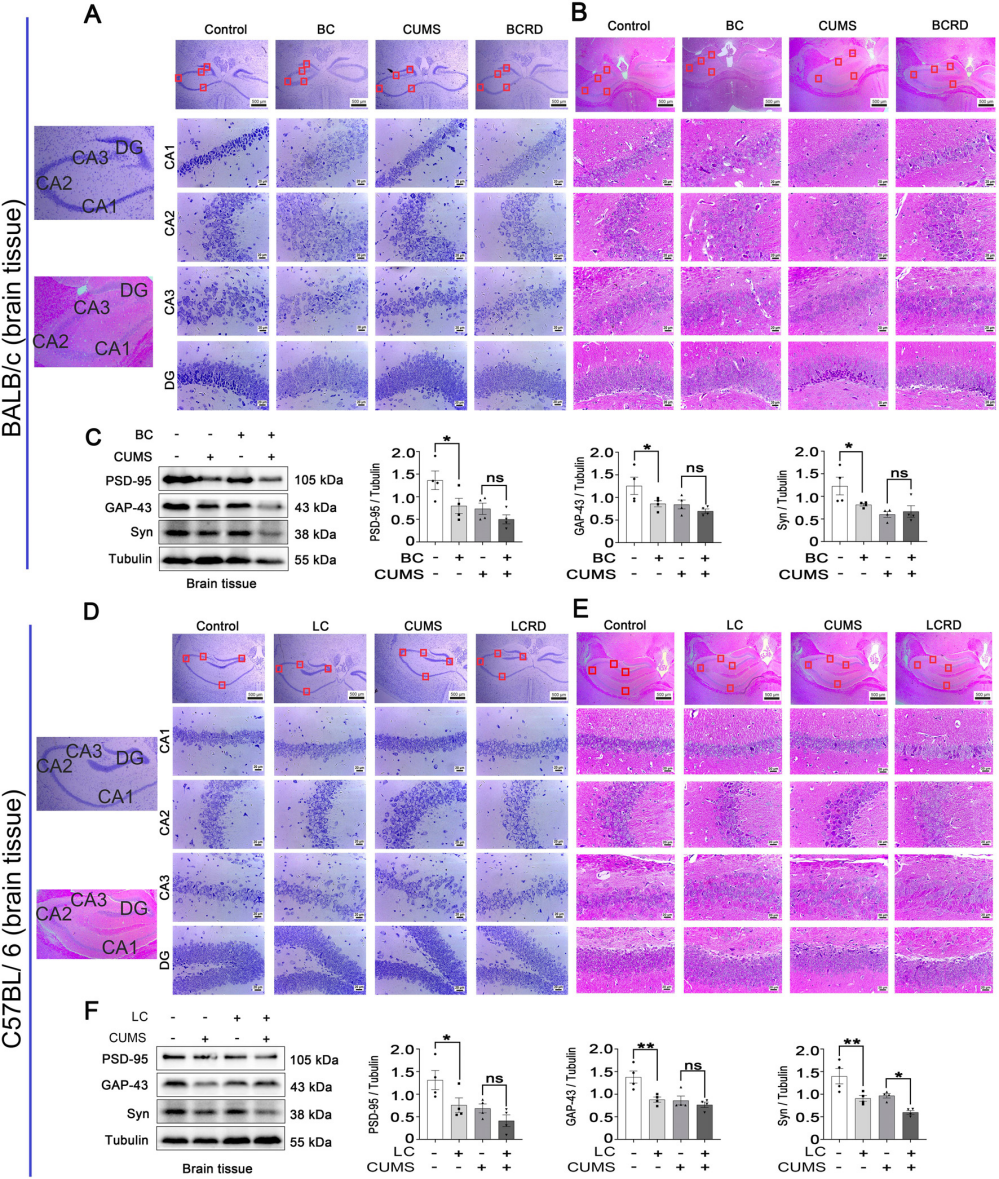
CUMS aggravated hippocampal neuron damage in tumor-bearing mice: **(A, D)** Nissl staining of the hippocampus in BALB/c mice and C57BL/6 mice. **(B, E)** H&E staining of the hippocampus in BALB/c mice and C57BL/6 mice. **(C, F)** Western blotting was used to detect the expression of brain plasticity enzymes. Data are represented as the mean value ± SEM (**C** and **F**, n= 4), * p<0.05, ** p<0.01. ns, no significant.

It is reported that hippocampal neurons play a crucial role in depression (5, 28), and Chronic stress causes pathophysiological changes in the hippocampus, which can induce depression (11, 29). As seen in **Figure 3**, the results of Nissl and H&E staining showed that hippocampal neurons were full and clear with a tight and neat cellular arrangement in the control group mice, the Nissl bodies were clear, and no obvious neuron degeneration. The hippocampus neurons were damaged, irregularly arranged, and sparsely distributed, with a widened interstitium in CA1, CA2, and CA3 regions in both CUMS group and two tumor groups (BC and LC). There was a significant decrease in the number of Nissl bodies and a tendency for them to spread to the outer layer. In addition, CUMS aggravated irregular arrangement and sparse distribution in CA2 and CA3 hippocampal regions in tumor-bearing mice, and Nissl bodies were absent and there were obvious neuronal “escapes” and ablation. These results suggest that CUMS aggravates hippocampal neuronal damage in tumor-bearing mice. There exists a complex relationship between cancer and neural remodeling and dysfunction. Western blotting results showed that PSD-95, GAP-43, and Syn were significantly lower in the BC and LC groups compared with the control group. Syn was significantly lower in the LCRD group compared with the CUMS group, no significant in the BCRD group compared with the CUMS group. PSD-95 and GAP-43 were not significant between the CUMS group and the BCRD and LCRD group (**Figures 3C, F**).

### 3.4 Chronic stress accelerated tumor tumorigenesis and metastasis

**Figure 4:**
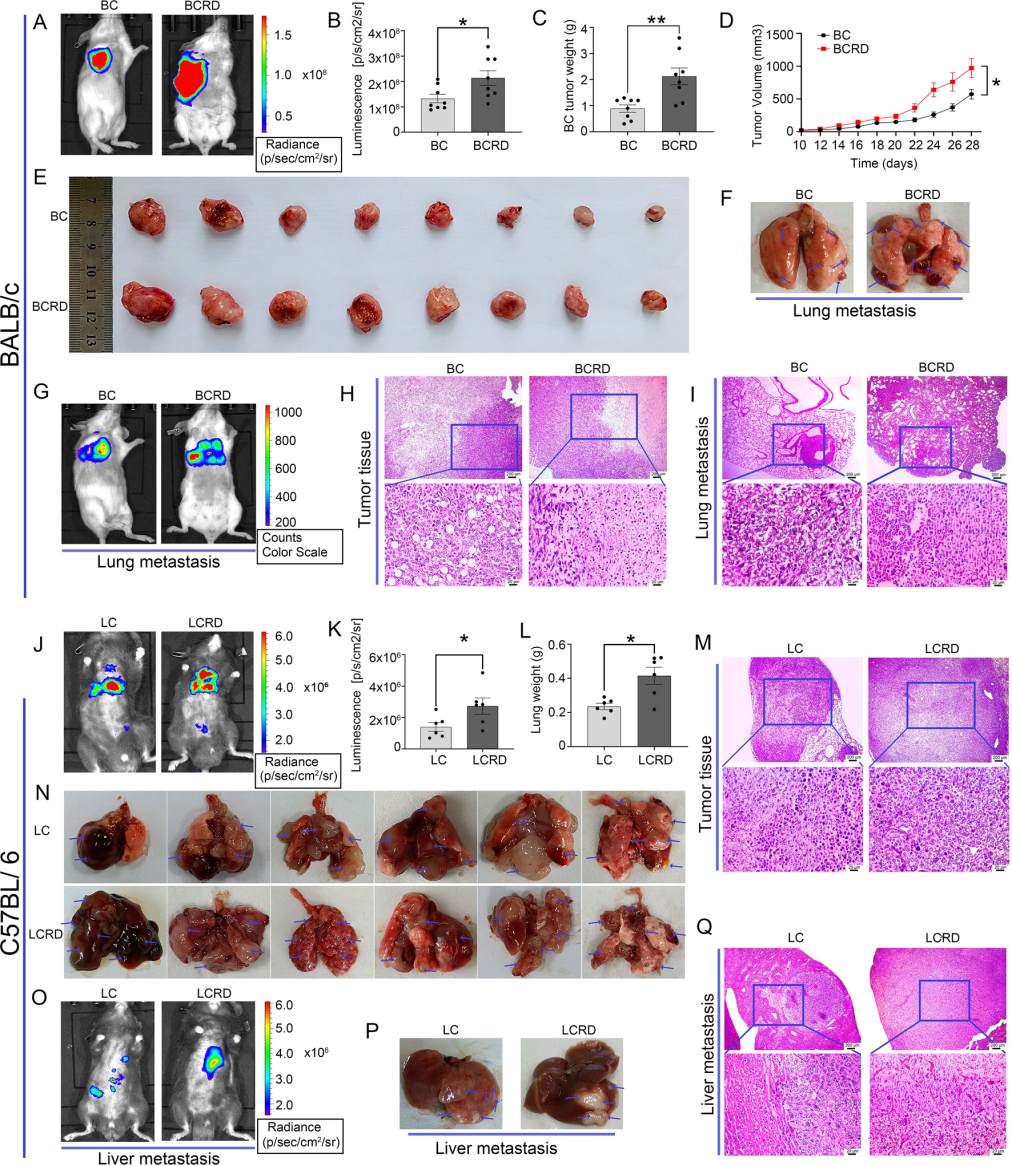
Tumor growth and metastasis were aggravated by chronic stress: **(A, E, F, G, J, N, O, P)** The representative pictures of vivo imaging assay and tumors in each group. **(B, C, D, K, L)** The fluorescence intensity values, Tumor weight, and growth curves. **(H, I, M, Q)** The representative pictures of tumor tissues and metastasis were stained with H&E staining. Data are represented as the mean value ± SEM (BALB/c: n= 8, C57BL/6: n= 6), * p<0.05, ** p<0.01.

Here, we used live mice imaging to track tumor growth and metastasis in both models of mice. Fluorescence intensity obtained by *in vivo* imaging, and tumor visual morphology *in vitro* showed that the tumors in tumor-bearing mice suffered from CUMS were more severe than those without CUMS (**Figures 4A, B, E, J, K, N**). At the same time, the volume of the mammary glands the weight of the BCRD mice, and the weight of the lungs of the LCRD mice increased significantly (**Figures 4C, D, L**). Tissue sections were prepared for pathologic analysis. H&E staining showed that more numerous and larger tumor foci with more dense tumor cells were observed both in BCRD and LCRD mice, and the core of the tumor showed a necrosis-like structure due to lack of nutrients. More scattered tumor-infiltrating cells were also observed in other residual normal tissue cells (**Figures 4H, M**).

The 4T1-Luc cells are reported to be a highly metastatic breast cancer cell line with a tendency to metastasize to the lung *in vivo* (30). This phenomenon has indeed been observed by *in vivo* imaging (**Figure 4G**). Furthermore, a greater number and size of metastases were discovered in BC mice exposed to CUMS (**Figure 4F**). The liver is a common priority metastasis site of lung cancer (31, 32). And the bioluminescence imaging results were confirmed (**Figure 4O**). As similar, more liver metastases were also observed in LCRD (**Figure 4P**). H&E staining results also showed that the cancer cell morphology in BCRD and LCRD mice metastases was more serious and deteriorated (**Figures 4I, Q**). The above results indicated that chronic stress was a high-risk factor for tumor metastasis.

### 3.5 Chronic stress has less influence on glycolysis within the brain tissues of tumor-bearing mice

Studies have shown that brain plasticity is one of the pathogenic mechanisms of depression, and glycolytic metabolism is closely related to synaptic plasticity (33–35).

**Figure 5:**
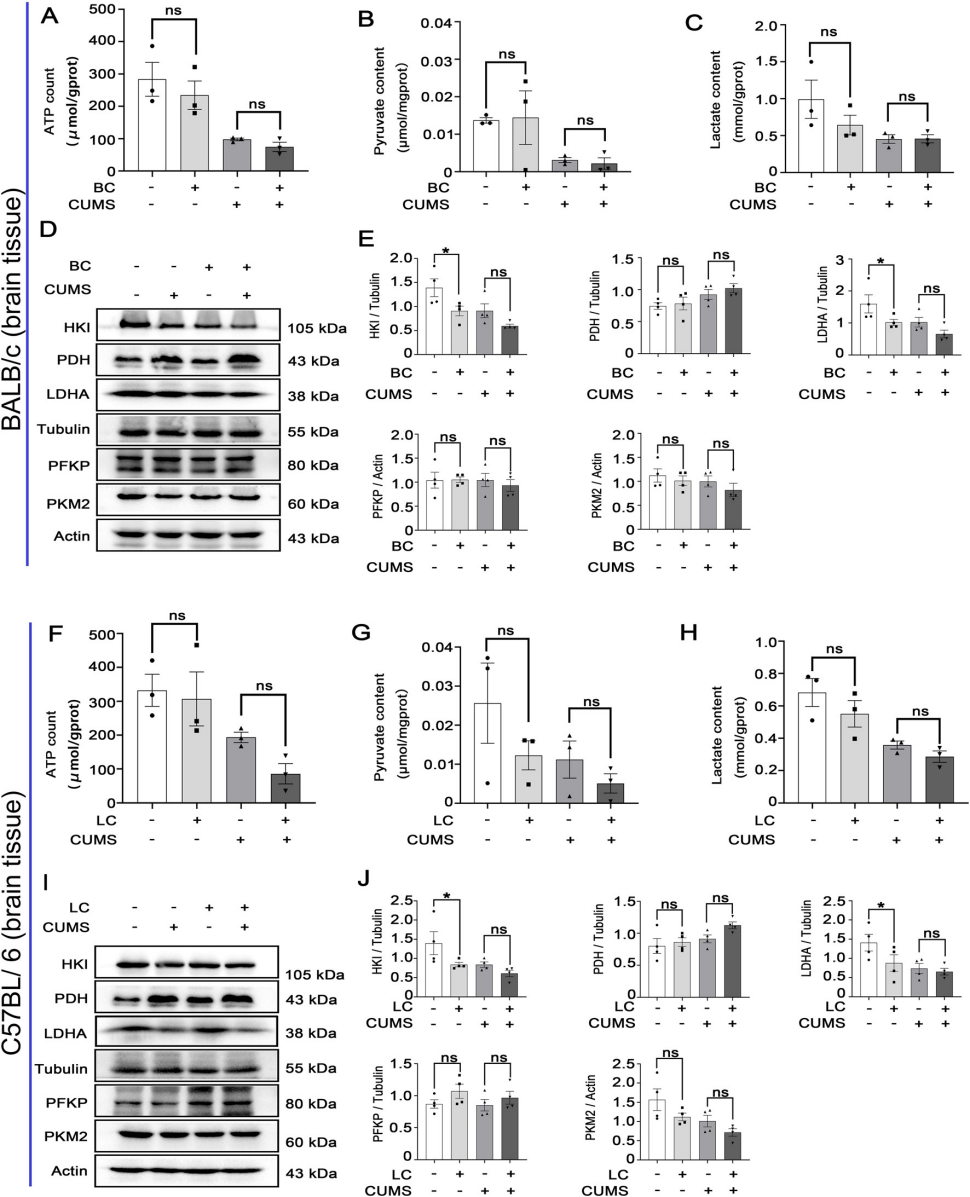
Chronic stress has less influence on glycolysis within the brain tissues of tumor-bearing mice: **(A-C, F-H)** Glycolytic metabolites (ATP, pyruvate, lactic acid) concentration. **(D, E, I, J)** The expression of glycolysis enzymes was detected with Western blot in mice brains. Data are represented as the mean value ± SEM, * p<0.05. ns, no significant.

In BALB/c mice and C57BL/6 mice, there were no significant differences in ATP, pyruvate, and lactic acid within the groups (**Figures 5A–C, F–H**). Glycolytic metabolic enzymes in the brain (HKI, PFKP, PKM2, PDH, and LDHA) were detected with the Western blot. Compared to the control group, the levels of HKI and LDHA were decreased in the BC group and LC group (**Figures 5D, E, I, J**). However, there were no significant changes observed in PDH, PFKP, and PKM2 expression in the CUMS group and BCRD group and LCRD group (**Figures 5D, E, I, J**).

### 3.6 Chronic stress augmented aerobic glycolysis within tumor tissues

**Figure 6:**
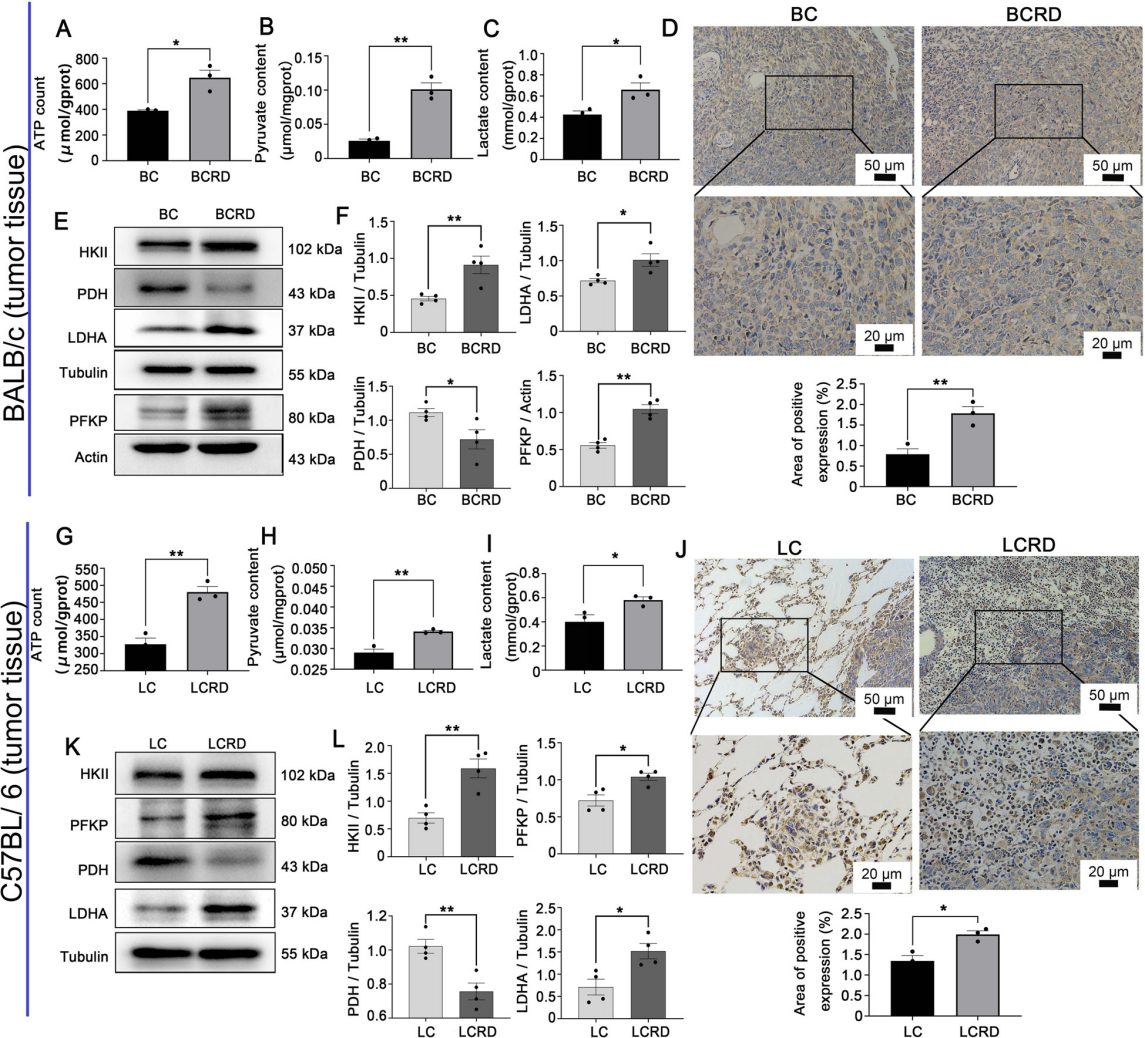
Aerobic glycolysis enhanced both in BCRD and LCRD. **(A-C, G-I)** Glycolytic metabolites in tumor tissue: **(D, J)** HKII distribution in tumor tissue was detected with IHC. **(E, F, K, L)** Western blotting was used to detect the expression of glycolysis enzymes in the tumor. Data are represented as the mean value ± SEM, * p<0.05, ** p<0.01.

Warburg effect is critical for the metabolic reprogramming of tumor cells (36, 37). The uptake of glucose was enhanced, leading to an increase in lactate production and extracellular acidification rates (26). Therefore, we investigated the regulatory role of CUMS in glycolysis. As shown in **Figure 6**, the tumor tissues of tumor-bearing mice (both BCRD and LCRD) pretreated by CUMS exhibited elevated levels of glycolysis products and key catalytic enzymes (**Figures 6A–C, E–I, K, L**). However, IHC detection results revealed the distribution pattern of HKII in BC and LC tissues is different (**Figures 6D, J**). The distribution of HKII in BC tissues is relatively uniform, and HKII is mainly confined to superficial tumor tissues, while HKII in LC tissues is diffusely dispersed in tumor cells and surrounding tissues of normal lung tissues.

## 4 Discussion

It has been reported that approximately 280 million people worldwide suffer from depression, and 800,000 of them die from depression each year (38–40). Nearly 80% of depression patients are not diagnosed in time (40). Animal and clinical studies have shown a link between emotional dysfunction and tumorigenesis (41). Approximately 20%-30% of patients with advanced cancer develop clinically significant depression, and 15% suffer from severe anxiety disorders (42). In this study, it was demonstrated that chronic stress can cause depression-like behavior and facilitated tumor genesis and metastasis of BC and LC, meanwhile, all the tumor-bearing mice exhibited depression-like behavior to different extents. In addition, Chronic stress is not only associated with depression but also with burnout and cognitive impairment (43). Among 102 cancer survivors aged 25-79 years, approximately one-third had cognitive failures in daily life (44–46). Our results suggest that chronic stress induces the production of depressive-like behaviors, reduces spatial cognition in mice, and induces mice to exhibit more severe depressive-like behaviors and cognitive dysfunction. These results are consistent with those reported in the literature (47).

Dysregulation of neuroplasticity and neuronal cell damage are thought to be key mediators in the pathogenesis of depression (35, 48, 49). Our results also confirmed that CUMS induced neuronal damage and synaptic plasticity reduction in mice, which is consistent with the literature (50, 51). Furthermore, cancer is intricately linked with neurological remodeling and dysfunction neurological-cancer interactions have the potential to modulate tumor growth, invasion, and metastasis dissemination (52). Tumor growth has been associated with substantial alterations in the hippocampus and a reduction in neuronal cell count, ultimately contributing to depressive symptoms (53). Notably, chronic stress is correlated with the activation of the neuroendocrine system, specifically the hypothalamic-pituitary-adrenal axis, and the sympathetic nervous system. Additionally, it leads to the release of stress hormones such as catecholamines and glucocorticoids (54). The microenvironment is changed by the disruption of stress, neurotransmitters, and immune cells and promotes tumorigenesis and progression through a variety of mechanisms (41). It was reported that the stimulation of β2-adrenergic receptors in PDAC cancer cells by noradrenaline induced by chronic stress leads to the production of autocrine and paracrine effects, thereby promoting tumor growth (55). Our findings also validate the notion that tumors can induce neuronal cell damage and diminish synaptic plasticity in mice. Chronic stress exacerbates neuronal impairment and reduces the survival rate of mice with tumors.

The Warburg effect is considered to be a critical element in the reprogramming of energy metabolism in malignant tumors (36). Chronic stress also plays an important role (10), and it promotes tumor growth and metastasis through multiple mechanisms (41). HKII is the first irreversible enzyme of glycolysis that inhibits the activity of the PDH complex by phosphorylating S1 of PDHA293 and promotes the Warburg effect (56). In addition to serving as an energetic metabolism substrate, lactate can be transported to neurons thereby sustaining neuronal function and exerting antidepressant effects (57, 58). Additionally, the augmented Warburg effect in the hippocampus contributes to enhanced synaptic plasticity (59). Most importantly, our results also show that chronic stress-induced higher expression of glycolytic metabolites in tumor tissue.

**Figure 7:**
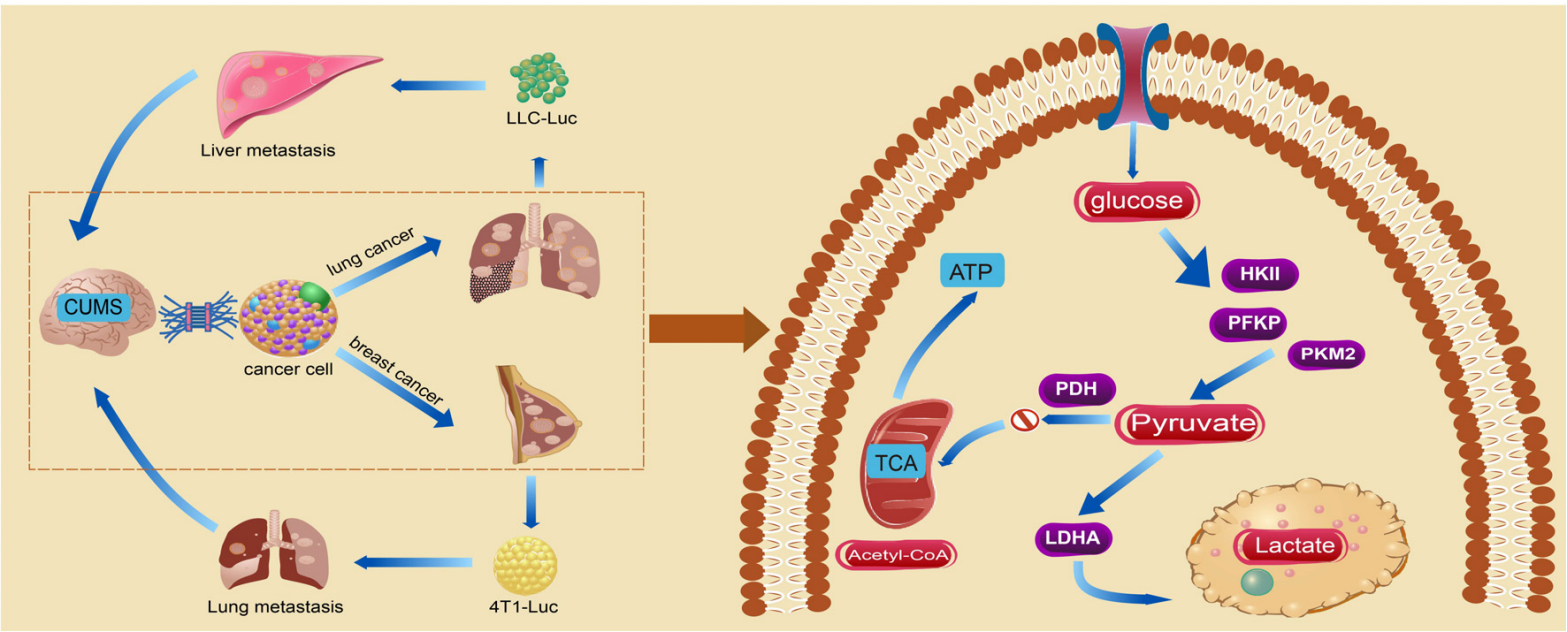
The chronic stress-induced depression-like behavior in mice exacerbated tumorigenesis and metastasis by augmenting glycolysis in tumor cells.

In conclusion, our findings consistently indicate chronic stress-induced anxiety-like and depression-like behaviors in mice, while also promoting the overproduction of lactic acid through increased aerobic glycolytic enzymes in tumor tissue. Extracellular acidification maintains the invasive growth of cancer cells and further exacerbates the progression and metastasis of BC and LC (**Figure 7**). The findings of this study contribute to a deeper understanding of the impact of metabolic remodeling on the pathogenesis of cancer-related depression. However, it was challenging to establish a strong connection between CUMS and glycolysis because of the small sample size, which limited the experiment’s findings to examining changes in glycolysis in tumor progression under chronic stress rather than delving into the underlying mechanisms in detail. As a result, future related studies should concentrate on assessing glycolysis’s significant contribution to the phenomenon of cancer-associated depression. In the meantime, because the cancer cell lines used in this study came from different species, different strains of mice were given different injections of cancer cells. It was discovered that this caused variations in the immune responses and stress levels in the male and female mice, which in turn led to variations in the model’s experiment outcomes. Consequently, we plan to inject the cancer cells into the same strains of mice in future related studies.

## Data Availability

The original contributions presented in the study are included in the article/supplementary material. Further inquiries can be directed to the corresponding authors.
